# The Dynamics of Nestedness Predicts the Evolution of Industrial Ecosystems

**DOI:** 10.1371/journal.pone.0049393

**Published:** 2012-11-19

**Authors:** Sebastián Bustos, Charles Gomez, Ricardo Hausmann, César A. Hidalgo

**Affiliations:** 1 Center for International Development, Harvard University, Cambridge, Massachusetts, United States of America; 2 Harvard Kennedy School, Harvard University, Cambridge, Massachusetts, United States of America; 3 Graduate School of Education, Stanford University, Stanford, California, United States of America; 4 Santa Fe Institute, Santa Fe, New Mexico, United States of America; 5 The MIT Media Lab, Massachusetts Institute of Technology, Cambridge, Massachusetts, United States of America; 6 Instituto de Sistemas Complejos de Valparaíso, Valparaíso, Chile; Northwestern University, United States of America

## Abstract

In economic systems, the mix of products that countries make or export has been shown to be a strong leading indicator of economic growth. Hence, methods to characterize and predict the structure of the network connecting countries to the products that they export are relevant for understanding the dynamics of economic development. Here we study the presence and absence of industries in international and domestic economies and show that these networks are significantly nested. This means that the less filled rows and columns of these networks' adjacency matrices tend to be subsets of the fuller rows and columns. Moreover, we show that their nestedness remains constant over time and that it is sustained by both, a bias for industries that deviate from the networks' nestedness to disappear, and a bias for the industries that are missing according to nestedness to appear. This makes the appearance and disappearance of individual industries in each location predictable. We interpret the high level of nestedness observed in these networks in the context of the neutral model of development introduced by Hidalgo and Hausmann (2009). We show that the model can reproduce the high level of nestedness observed in these networks only when we assume a high level of heterogeneity in the distribution of capabilities available in countries and required by products. In the context of the neutral model, this implies that the high level of nestedness observed in these economic networks emerges as a combination of both, the complementarity of inputs and heterogeneity in the number of capabilities available in countries and required by products. The stability of nestedness in industrial ecosystems, and the predictability implied by it, demonstrates the importance of the study of network properties in the evolution of economic networks.

## Introduction

One of the best-documented findings of biogeography is that rare species inhabit predominantly diverse patches, while ubiquitous species tend to inhabit both, diverse and non-diverse locations [1]–[4]. In ecology, the term *nestedness* is used to refer to this feature, which has been observed numerous times in geographic patterns [1]–[4] and mutualistic networks [5]–[8]. In the case of mutualistic networks, nestedness implies that ecosystems are composed of a core set of interactions to which the rest of the community is attached [5]. The nestedness of interaction networks also implies that specialist species interact mostly with generalist species, and because generalist are less fluctuating [9], nestedness can help enhance the survival of rare species [10]. Nestedness has also been shown to enhance biodiversity [11] and overall ecosystem stability [12], and therefore, it is considered an important structural property of interaction networks in ecology.

Nestedness, however, is a general network measure that can be used to characterize non-biological ecosystems, such as global and local economies. In fact, in the past, the nestedness of economic systems has been described for interaction networks, connecting industries to other industries, such as the input-output matrices introduced half a century ago by Leontief [13], or the supply relationships in the New York Garment industry [14], [15].

Here, we study the dynamics of economic geographic, instead. We look at the presence and absences of industries across a wide range of locations and show that (i) nestedness tends to remain stable; (ii) it can be used to predict the location of industrial appearances and disappearances; and (iii) can be accounted for by a simple model.

In recent years, the structure of industry-location networks has received a wide range of attention. A country's level of income is tightly connected to the mix of products that they export [16]–[18], as measured by their Economic Complexity Index or ECI [16], [17]. The ECI is a structural measure of the network connecting countries to the products that they export that estimates the amount of productive knowledge embedded in a country [16] from information on who exports what. Countries that have an income that is lower than what would be expected from their ECI, such as China, India and Thailand, tend to grow faster than those that have an income that exceeds what would be expected from their current level of economic complexity, such as Greece and Portugal [16], [17]. Hence, what countries export, as proxied by the ECI, is a strong leading indicator of economic growth.

In the past, the network connecting countries to the products that they export has been used to identify related varieties [19]–[21]. Here, products that tend to collocated, or co-exported, are connected with a strength that grows with the probability of co-export. Colocation networks, like the product space [20], have been used to show that the productive structure of countries, and regions, evolve as these move from the products that they do to others that are close by in this network. The use of colocation data provides an alternative to more data intensive methods, such as networks connecting industries based on labor flows, labor similarities [22] or plant level data [23]. This is because labor and plant level data lacks standardized international coverage and therefore cannot be used for international comparisons.

The evolution of a country's product mix, however, is highly path dependent [16], [20]. Here, we look at the nestedness of the industry location network and show that deviations from nestedness can help predict these path dependencies for both, industrial appearances and disappearances. These predictions add to our ability to explain the evolution of a country's product mix, and therefore, variations in cross-country levels of income. Moreover, we show that the high level of nestedness observed in the data can be reproduced using a simple model when we assume that the heterogeneity of capabilities available in a country, or required by a product, is large.

The paper is structured as follows. First, we study the nestedness of the industry locations matrix and find it to be highly stable over time. We do this by using Almeida-Neto et al's NODF [24], [25] (and Atmar and Patterson's Temperature metric [26], [27] in the SM). We asses the stability of nestedness by comparing it with both, static and dynamic null models, showing that the observed level, and stability of the network's nestedness, is larger than what would be implied by these null models.

Next, we show that deviations from nestedness are associated, respectively, with increases and decreases in the probability that an industry will appear or disappear at a given location. Finally, to provide an explanation of the observed phenomena we generalize the model recently introduced by Hidalgo and Hausmann [16], [28] to show that this model can account for both, the high level of nestedness values, and their stability.

Together, these results illustrate the relevance of nestedness for the evolution of industrial ecosystems and shows that a simple model can account for the high level of nestedness observed in economic networks.

## Data and Methods

The ideal data to study the patterns of economic geography would consist of plant level information, collected for all countries, with high spatiotemporal resolution, and following a disaggregate standardized classification covering all economic sectors. Unfortunately, such data is not available. Instead, we use yearly trade data connecting 114 countries to 772 different products. Here, products are classified according to the SITC-4 rev2 classification. We use data from 1985 to 2009 to approximate the evolution of the global patterns of production. Going forward, we refer to this as the country-product network. We consider a country to be connected to a product if that country's exports per capita are larger than 25% of the world's exports per capita in that product for at least five consecutive years. These thresholds reduce the noise in the country product data coming from re-exports and helps make sure that a country is connected to the products that they export substantially and consistently. In Materials S1 we check for the robustness of our results by using a different definition of presences and absences based on Balassa's [29] Revealed Comparative Advantage (RCA), and find the results to be robust to this alternative definition of presences.

We note two important limitations of international trade data. First, it does not include products that are produced and consumed domestically. This is because it only considers a product once it has crossed an international border. Second, trade data is limited to goods, and therefore does not include any data on services. Despite these limitations, trade data is good for international comparisons because it is collected in a standardized classification that makes data for different countries comparable.

At the domestic level we use information on the tax residence of Chilean firms collected by Chile's *Servicio de Impuestos Internos* (SII), which is the equivalent of the United States Internal Revenue Service (IRS). Going forward, we refer to this dataset as the municipality-industry network. The municipality-industry network contains information on 100% of the firms that filed value-added and/or income taxes in Chile between 2005 and 2008. This data comprises firms from all economic sectors, whether they export or not, and whether they produce goods or services. The municipality-industry network consists of the universe of Chilean firms (nearly 900,000), which are classified into 700 different industries and assigned to each of Chile's 347 municipalities. Here we consider an industry to be present in a municipality if one or more firms, filing taxes under that industrial classification, declare that municipality as their tax residency.

Finally, we note that the Chilean tax data has the limitation that the tax residency of a firm can differ from the location of all of its operations. Going forward, we take the fact that our results hold in both, international trade and domestic tax data, as an indication that they are not driven by the limitations of these datasets and that they represent a natural characteristic of the economic networks underlying them. For more details on both datasets see the SM.

## Results

**Figure 1:**
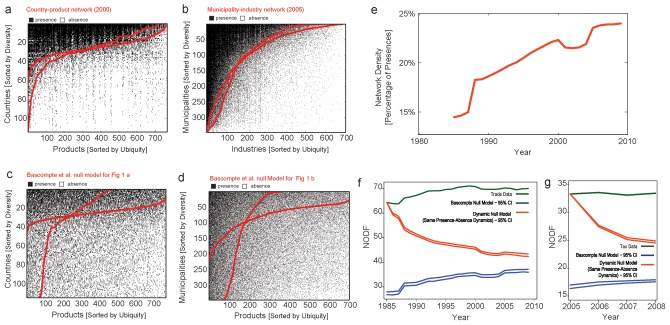
The nestedness of international and domestic economies. **a** Country-product network for the year 2000. **b** Municipality-industry network for the year 2005. **c** Bascompte et al. null model for the matrix shown in **a**. **d** Bascompte et al. null model for the matrix presented in **b**. In **a–d** red lines indicate the diversity of a location and the ubiquity of an industry (see full text for details). **e** Evolution of the density, or fill, of the country-product network between 1985 and 2009. **f** Evolution of the NODF of the country-product network between 1985 and 2009 (green), its corresponding Bascompte et al. null model (blue, upper and lower lines indicate 95% conf. intervals), and that of a matrix that started identical to that for 1985, but that was evolved by considering an equal number of appearances and disappearances than in the original data (red, upper and lower lines indicate 95% conf. interval). **g** Same as **f** but for the municipality-industry network (see SM for results with Atmar and Patterson's temperature metric).

Figures 1 **a** and **b** show the matrices of the country-product and the municipality-industry networks (Respectively NODF = 70.81 and NODF = 83.35. We note that NODF = 100 indicates perfect nestedness and NODF = 0 indicates no nestedness, [30]). Here, the red lines indicate the diversity of each country and the ubiquity of each product -the number of locations where it is present- (see SM). These lines are used as a guide to indicate where presences would be expected to end if the nestedness of these networks were to be perfect. They can be thought of a simplified extinction line [27]. Figure 1 **c** and **d** show their corresponding Bascompte et al. null models [5]. In the Bascompte et al. null model, the probability to find a presence in that same cell of the matrix is equal to the average of the probability of finding it in that row and column in the original matrix. The figures show that nestedness of the original networks is clearly larger than that of their respective null models, showing that industrial ecosystems are more nested than what would be expected for comparable networks (respective null model NODF of 35.0±0.6 and 46.5±0.3, errors are 99% confidence intervals calculated from 100 implementations of the null model).

Next, we study the temporal evolution of nestedness. In the case of the country-product network, where a larger time series is available (1985–2009), the percentage of presences almost doubled during the observation period (Figure 1 **e**), going from less than 15% to nearly 25%. In the case of the municipality-industry network, presences went up from 22.9% to 25.7% between 2005 and 2008. The nestedness of both, the country-product and the municipality-industry networks, however, remained relatively stable during this period as measured by NODF (green lines in Figure 1 **f**–**g** and SM).

We test the constancy of these networks' nestedness by comparing them with two null models. The first one is an ensemble of null models [5] calculated for each respective year (blue lines in Figure 1 **f**–**g**). This shows that the nestedness of the empirical networks is always significantly higher than their randomized counterpart. Then, we show that a network subject to the same exact turnover dynamics would have lost its nestedness during the observation period. We do this by starting with the empirically observed network and simulate its evolution by sequentially adding and subtracting a number of links equal to the one gained or lost by the original network. We do this following the probability distributions defined by the Bascompte et al null model [5] to make sure that these additions and subtractions keep the degree sequence of the network close to the original one. Otherwise, the lost of nestedness could be a consequence of changes in the underlying distributions. This dynamic null model represent a strong control, since it preserves the exact density of the network and also its turnover dynamics, as the number of links that appeared and disappeared each year, in each country, and for each product is exactly that observed in the original data. The dynamic model, however, does not preserve nestedness, showing that its stability comes from the specific way in which links appear and disappear from the network, and not due to a more trivial dynamics. In fact, when the appearance and disappearance of the links are chosen differently, the nestedness of the network quickly evaporates (red line in Figure 1 **f–g**). This allows us to conclude that the stability of nestedness observed in these networks is higher than what would be expected from a null model with the same general turnover dynamics.

Could the stability of nestedness be used to predict appearances and disappearances? In the past, nestedness has been used to make prediction of the biota available in ecological patches, albeit not in economic networks [2], [31]. For the country-product network we consider as an appearance an increase in exports per capita from less than 5% of the world average to more than 25%. To make sure that we are capturing structural changes and not mere fluctuations, we ask the increase in exports per capita of a country to be from less than 5%, for five consecutive years, to more than 25% sustained for at least 5 years. Hence, our final year of observation is 2005. Conversely, we count disappearances as a decrease in exports per capita of a country from 25% or more of the world's average to 5% or less (also sustained for at least 5 years). For the municipality-industry network we count appearances as changes from zero industries to one or more, and disappearances as changes from one or more industries to zero.

**Figure 2:**
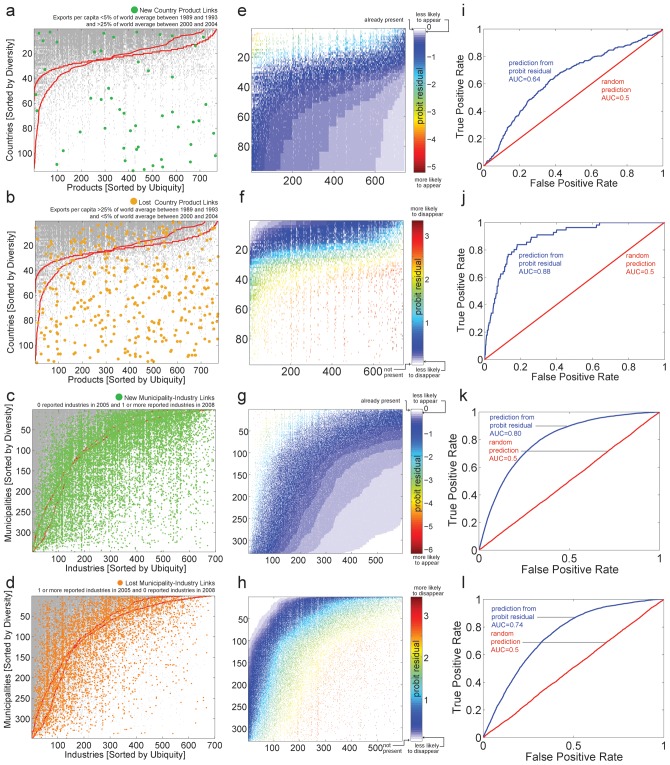
Nestedness predicts appearing and disappearing industries. **a** The country-product network for the year 1993 is shown in grey. Green dots show the location of industries that were observed to appear between 1993 and 2000. **b** Same as **a**, but with the industries that disappeared in that period shown in Orange. **c** The municipality-industry network is shown in grey and green dots show the location of industries that were observed to appear between 2005 and 2008. **d** Same as **c**, but with the industries that disappeared in that period shown in Orange. **e–h** Deviance residuals of the regression presented in (1) applied to the presences-absences shown in **a–d**. **i–l** ROC curves summarizing the ability of the deviance residuals shown in **e–h**, to predict the appearances and disappearances highlighted in **a–d**.

Figure 2 **a–d** visualizes the position in these networks' adjacency matrices of the industries that were observed to appear (green) and disappear (orange) in the intervening period. We predict these appearances and disappearances by fitting each observation in the industry-location network using a probit model that considers information on the diversity of the location and the ubiquity of the industry for the initial year (see SM). This represents a parameterization of nestedness and is similar to previous approaches that have used nestedness to make predictions [2], [31]:
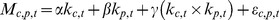
(1)Here 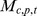 is the industry-location network's adjacency matrix, 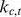
is the diversity of location *c* at time *t* (defined as its degree centrality or 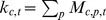), 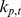 is the ubiquity of product *p* at time *t* (defined as its degree centrality or 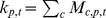), and where we have also added an interaction term taking the product between diversity (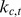) and ubiquity (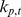). The error term is represented by 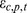. We find that all coefficients are highly significant, meaning that a model that would only consider diversity or ubiquity, or both of them without an interaction term, would not be as accurate.

In general, we find that the probit regression accurately explains presences and absences (average Efron's pseudo-R^2^ = 0.53±0.02 for the country-product network and 0.54±0.01 for the municipality-industry network). Here, however, we use the deviance residuals of this regression to predict future appearances and disappearances. Negative residuals, represent unexpected absences [2] and are used to rank candidates for new appearances. Positive deviance residuals, on the other hand, represent unexpected presences [2] and are used to rank the likelihood that an industry will disappear in the future. (Figures 2 **e**–**h**).

But how accurate are these predictions? We quantify the accuracy of predictions by using the area under the Response Operator Characteristic curve or ROC curve [32], [33]. An ROC curve plots the true positive rate of a prediction as a function of its false positive rate. The Area Under the Curve, or AUC, is commonly used to measure the accuracy of the prediction criterion [32], [33]. A random prediction will find true positives and false positives at the same rate, and therefore will give an AUC of 0.5. A perfect prediction, on the other hand, will find all true positives before hitting any false positive and will be characterized by an AUC = 1. Figures 2 **i**–**l** show the ROC curves obtained when the appearances and disappearances shown in Figures 2 **a**–**d** are predicted using the deviance residuals obtained from (1) for data on the initial year. In all cases, the ROC curves of these predictions (in blue), have an area that is significantly larger than the one expected for a random prediction (in red), showing that nestedness can help predict which links in these industry-location networks are more likely to appear or disappear.

**Figure 3:**
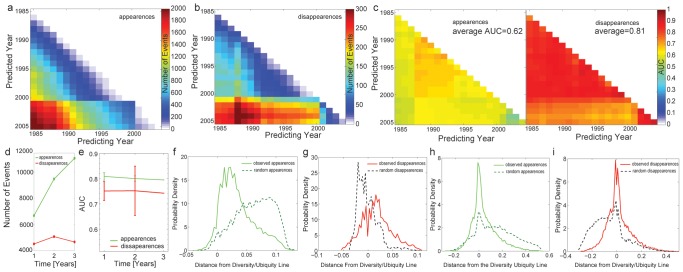
Predicting appearances and disappearances using nestedness. **a** Number of appearances for every pair of years in the country-product network. **b** Number of disappearances for every pair of years for the country-product network. **c** Accuracy of the predictions for each pair of years measured using the Area Under the ROC Curve (AUC). **d** Average number of appearances and disappearances for the Chilean data (error bar smaller than symbol). **e** Average accuracy of the predictions for the municipality-industry network. Error bars indicate 99% confidence intervals. **f** Distribution for the distance to the diversity-ubiquity line obtained for the observed appearances and for an equal number of random appearances. **g** Same as **f** but for disappearances. **h** Same as **f**, but for the municipality-industry network. **i** Same as **h** but for disappearances.

Finally, we extend this analysis to all pairs of years. Figures 3 **a** and **b** show the number of events (appearances or disappearances) for each pair of years for the international trade data. As expected, there are fewer events for pairs of years that are close by in time. Also, we note that the number of appearances is larger than that of disappearances, a fact that is consistent with the observed increase in the density of the network. Figure 3 **c** shows the AUC value obtained for each pair of years, showing that for the country product network, disappearances (Fig. 3 **b**) are predicted much more accurately than appearances.

The time series data available for Chile's municipality-industry network is much more limited. Hence, we show the average number of events (Figure 3 **d**), and the average AUC for networks separated by a given number of years (Figure 3 **e**). Here, we find that predictions of appearances and disappearance are both remarkably strong, and that there is no statistically significant difference in the predictability of both kinds of events.

To conclude this section, we look at the position in the network's adjacency matrix of appearances and disappearances. If the stability of nestedness is related to the location in this matrix of industrial appearances and disappearances, then appearances should be closer to the diversity-ubiquity line than random appearances. By the same token, disappearances should be farther away. For each event, we estimate its distance to the diversity and the ubiquity lines illustrated in figures 1 **a**–**d** and figures 2 **a**–**d** using,
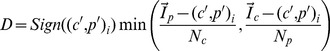
(2)


Here 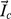 and 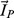 are respectively the lines of diversity and ubiquity (i.e. the red lines in Figure 1 a–d), 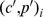 is the position in the adjacency matrix of the *i^th^* event, and *N_c_* and *N_p_* are respectively the number of locations and industries in the network. We use *N_c_* and *N_p_* to normalize the maximum possible vertical and horizontal distances to 1 and thus make sure that the measure is less sensitive to the rectangularity of the different matrices. The ∥ operator represents the Euclidean distance and 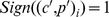 if the position of the event is outside of the nested area defined by both 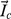 and 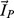 and −1 otherwise (see SM). As a benchmark comparison we consider an equal number of appearances and disappearances, but draw these from a random set of eligible positions in the adjacency matrix.

Figures 3 **f**–**i** compare the distributions of distances (*D*) with those associated with an equal number of random appearances or disappearances. We find that appearances tend to lie significantly closer to the diversity/ubiquity lines than what would be expected for an equal number of random events (ANOVA F = 59,935, p-value = 0 for the country-product network and ANOVA F = 10895 p-value = 0 for the municipality-industry network). In the case of disappearances, the opposite holds true. The observed appearances tend to be mostly located outside of the nested area defined by the diversity/ubiquity lines. Our random expectation, however, would be for disappearances to come mostly from the highly populated area inside the diversity/ubiquity lines. Once again, differences between observations and null model expectations are highly significant for both networks (ANOVA F = 6246 p-value = 0 for the country-product network and ANOVA F = 6463 p-value = 0 for the municipality-industry network).

Finally, we show that a modified version of the neutral development model introduced in [17], and solved analytically in [28], can be used to explain both, the observed level of nestedness and its stability. This neutral development model consists of three simple assumptions;

- Products require a set of non-tradable inputs, or capabilities, to be produced.
- Locations are characterized by a set of capabilities.
- Locations can only produce the products for which they have all the required capabilities.

The model is formalized by introducing three mathematical objects: two matrices and one operator. *P_pa_* is a matrix that is 1 if product *p* requires capability *a,* and 0 otherwise. *C_ca_* is a matrix that is 1 if location *c* has capability *a*, and zero otherwise. Finally (iii) provides a way of mapping *C_ca_* and *P_pa_* into *M_cp_*, since it implies that *M_cp_* = 1 if the set of capabilities required by a product is a subset of the capabilities available in a location. Mathematically (iii) can be expressed as the following operator:
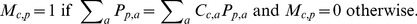
(3)


More details about the model can be found in [28].

To compare the model to the data we need to assume the form of *C_c,a_* and *P_p,a_*. In [28] the model was solved analytically by assuming that both, *C_c,a_* and *P_p,a_* were random matrices. This means that each location has a capability with probability *r* and that products require a capability with a probability *q*. From this we can trivially deduce that the number of capabilities available in a random country, or required by a random product, follows a binomial distribution. Because of this, we call this implementation of the neutral model: the binomial model. The third and final parameter that needs to be specified is the number of capabilities required by a product (*N_a_*). This is because the number of locations *N_c_*, and the number of products *N_p_*, is fixed to match the number of locations and products observed in the data.

Effectively, the binomial model has two free parameters. This is because it is always possible to determine *r*, *q* or *N_a_* once the fill of the *M_c,p_* matrix is known. The binomial model has been shown to reproduce the distribution of diversities, ubiquities, co-exports, and the relationship between diversity and ubiquity of the country-product network using *N_a_* = 80, *r* = 0.87 and *q* = 0.18. In addition to the binomial model we consider an alternative form that has the same number of parameters. We call this the uniform model, since in this case the number of capabilities that a country has is distributed uniformly between 0 and *R* and the number of capabilities that a product requires is distributed uniformly between 0 and *Q*. Hence, in this model country *c* has a capability *a* with probability equal to *r_c_* = min(1,*R*×*c*/*N_c_*). We take the minimum to ensure *r_c_* is upper bounded by 1. In the uniform model, allowing values of *R* larger than one allows having a small number of fully diversified countries.

**Figure 4:**
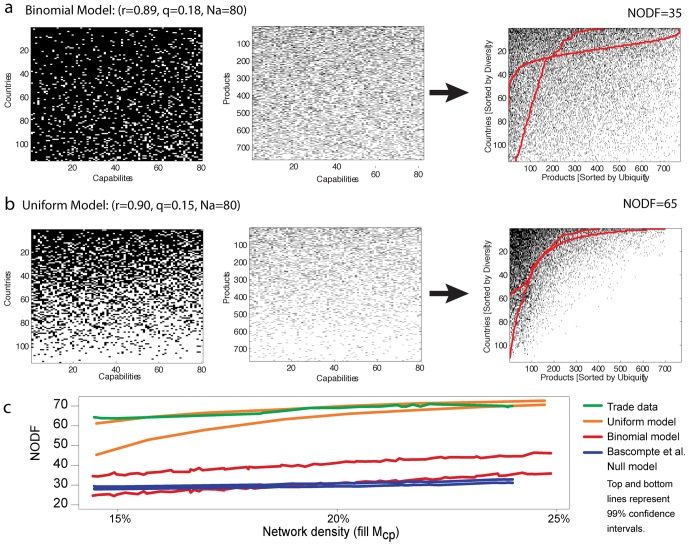
Modeling nestedness. **a** Illustration of the binomial model. From left to right; *C_ca_*, *P_pa_* and the resulting *M_cp_*. **b** Illustration of the uniform model. From left to right *C_ca_*, *P_pa_* and the resulting *M_cp_*. **c** NODF as a function of matrix fill for the country-product network (green), the uniform model (orange), the binomial model (red), and the Bascompte et al null model (blue).

Figures 4a and 4b illustrate the binomial model and the uniform model, respectively. For both models, we show their respective *C_c,a_* and *P_p,a_* matrices together with their resulting country-product network *M_c,p_*. We find that in both cases the resulting *M_c,p_* matrices are significantly more nested than the null model, yet the nestedness emerging from the uniform model is considerably larger, resembling closely the values observed for the country-product network. This comes from the fact that countries with a diverse capability endowment are likely to make a wide range of products, whereas countries with few capabilities will only be able to make those products that require few capabilities. This last observation is implied by assumption (iii), and is therefore true for both, the binomial and the uniform model. Yet, the large degree of heterogeneity among countries and products present in the uniform model enhances the nestedness implied by the complementarity assumption.

Figure 4 c compares the nestedness of the country-product network with the one found for the neutral models and null model. Here we plot nestedness as a function of the fill of the network since this is a good proxy for time and the neutral models and null model do not have an explicit time dimension. We implement this comparison by generating an initial *P_p,a_* matrix that is kept constant during the procedure. In the binomial model we choose *q* = 0.18, and for the uniform model we take *Q* = 0.21. We interpret this as an assumption that productive technologies change slowly during the time frames considered, and therefore, the increases in diversification observed in the empirical network comes from locations catching up to produce the products that more diversified locations were already making. To create *M_c,p_*, we generate 100 *C_c,a_* matrices for 200 different values of *r* and *R*. For the binomial model we consider values of *r* between 0.9 and 0.95, while for the uniform model we consider values of *R* between 0.9 and 1.07. In both cases we set the total number of capabilities in the system to *N_a_* = 80. These values are chosen to ensure that the fills of the modeled *M_c,p_* matrices are close to the ones observed in the original data. The analysis shows that the nestedness of the *M_c,p_* matrices implied by the neutral model matches the ones observed in the economic networks only for the uniform model. In the context of assumptions (i)–(iii), we interpret this result as evidence that heterogeneity in the distribution of capabilities available in a country, or required by a product, are needed to generate the high levels of nestedness observed in these economic networks.

## Discussion

In this paper we showed that industry-location networks are nested, just like industry-industry networks [13]–[15], or their biological counterparts [1]–[4], [26], [27]. Using time series data for both, international and domestic economies, we showed that the nestedness of these networks tends to remain constant over time and that this empirical regularity can be used to predict the pattern of industrial appearances and disappearances over time. Moreover, we showed that the high level of nestedness observed in the world can be accounted for by a simple model, but only if we assume a relatively large degree of heterogeneity in the number of capabilities present in a country or required by a product.

The strong link between biological and industrial ecosystems opens a variety of questions. First, is the geographical nestedness described in this paper a consequence of industry-industry nestedness, or are these independent phenomena? Second, are the mechanisms generating nestedness at the global level the same that generate nestedness at the national level?

In this paper we showed that the geographical nestedness of industries holds at both, the global and at the national scale. This is certainly not the case for biological ecosystems, since the biota of the artic is not a subset of that of the rain forest. The fact that the nestedness of industrial ecosystems holds at scales as large as that of the world economy suggests that the coupling between international economies is strong. This highlights the importance of understanding the global economy as a unified ecosystem, since after all, its nestedness suggests that it appears to be working as one.

The predictability implied by nestedeness, on the other hand, has important implications in a world where income is connected to the mix of products that a country makes [17], [18]. Ultimately, the dynamics implied by nestedness could represent a fundamental constraint to the speed at which international incomes could either converge or diverge.

More research will certainly need to be done on both, the causes of the structures and the time patterns that were uncovered in this paper. This will require strengthening the bridge between the natural and social sciences because, if there is something that the nestedness of economies show, is that humans tend to generate patterns in social systems that strongly mimic those found in nature [34], [35].

## Supporting Information

Materials S1 Additional information on the data and methods used throughout the manuscript.
(DOCX)
