# Supplementary material for: The Dynamics of Nestedness Predicts the Evolution of Industrial Ecosystems
Source: PLoS One. 2012 Nov 19;7(11):e49393. doi: 10.1371/journal.pone.0049393 (PMC3501524; doi:10.1371/journal.pone.0049393)
Supplement: Materials S1 — Additional information on the data and methods used throughout the manuscript. (DOCX) [file pone.0049393.s001.docx]

**­*SUPPLEMENTARY MATERIAL FOR:***

**­­The Dynamics of Nestedness Predicts the Evolution of Industrial Ecosystems**

**Sebastián Bustos^1,2^, Charles Gomez^3^ & Ricardo Hausmann^1,2,4^,César A. Hidalgo^1,5,6,^**^†^

^1^ Center for International Development, Harvard University, Cambridge, Massachusetts, United States of America
^2^ Harvard Kennedy School, Harvard University, Cambridge, Massachusetts, United States of America

^3^ Program on Organization Studies, Stanford University
^4^ Santa Fe Institute, Santa Fe, New Mexico, United States of America

^5^ The MIT Media Lab, Massachusetts Institute of Technology, Cambridge, Massachusetts, United States of America

^6^ Instituto de Sistemas Complejos de Valparaíso, Valparaíso, Chile

^†^ hidalgo@mit.edu

TABLE OF CONTENTS

DATA DETAILS: 17

International Trade Data: 17

Domestic Tax Data: 18

PRESENCE-ABSENCE MATRIX DEFINITION: 18

*NESTEDNESS METRICS: TEMPERATURE AND NODF* 18

*ATMAR AND PATTERSON’S TEMPERATURE MEASURE* 19

*Almeida-Neto et al.’s NODF Measure* 20

NULL MODELS 22

Static Null Model (Bascompte et al.) 23

Dynamic Null Model 23

DIVERSITY AND UBIQUITY LINES, AND DISTANCE OF EVENTS 23

ROBUSTNESS CHECKS FOR NESTEDNESS 24

# DATA DETAILS:

## International Trade Data:

The international data set is a merge of two data sources: The Feenstra et al. (2005) data set, which has data for the years prior to 2000, and the UN Comtrade database (comtrade.un.org), which we used for the period going from 2001 to 2009. Both dataset follow the product classification established by the Standard International Trade Classification (SITC) revision 2^[[1]](#footnote-1)^. In the UN Comtrade dataset we associated countries to products according to what was reported as exports to the WLD category (World). For the products in which no exports to WLD (World) was found, exports were reconstructed using the reports from importing countries, when available, and by aggregating the reported bilateral exports of the exporting country as a last resource. We prioritize imports over exports because imports tend to be more tightly controlled than exports.

While the Feenstra et. al (2005) data set contains trade starting 1962, we chose 1985 as our starting year because there are several reclassifications of the data that affect their reliability for previous years (see SM2 Data Continuity). Since presences are averages over 5 years, the first year that is included in our dataset is 1981 (in the counting of presences for 1985).

We find, however, that international trade data is characterized by a nested matrix even for the years that we do not include in this paper. Figure SM1 shows the Temperature and NODF calculated for all years. Our choice to restrict the number of years in the dataset was performed to reduce the number of false appearances and disappearances that could be introduced by reclassifications of the SITC categories.


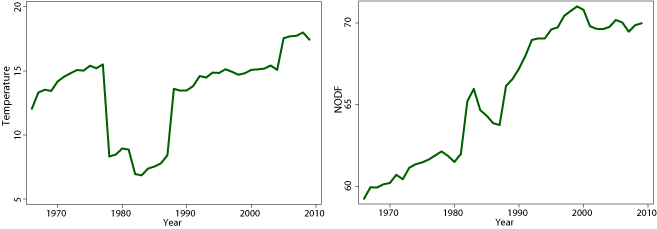

**Figure SM1** The nestedness of international trade data from 1966 to 2009. Note the range of the y-axis.

Finally, we restrict the sample of countries for all those that have a population of at least 1.25 million in the year 2000. We also remove countries from the Former Soviet Union (FSU), because this countries lack data for the 1980's, and have noisy data for the early 1990's. For Germany, we use data on West Germany for the years prior to 1992.

The final dataset consists of 114 countries and 775 products, classified according to the SITC4 rev2 classification (<http://reportweb.usitc.gov/commodities/naicsicsitc.html>). The datasets includes only tradable products, from raw materials and agriculture, to manufactures and chemicals.

## Domestic Tax Data:

The domestic data for Chile consists of a matrix indicating the number of firms from a given industry in each municipality. The data has records for the year 2005, 2006, 2007 and 2008 and is based on the fiscal residence of each firm (it is hence a firm, and not a establishment level dataset). The number of firms reported for each year is shown in table 1.

| Year | Number of Firms |
| --- | --- |
| 2005 | 862,405 |
| 2006 | 876,948 |
| 2007 | 891,383 |
| 2008 | 899,156 |

Table 1: Number of Firms in the Chilean Tax Data

These data contains information on the universe of Chilean firms and includes firms from all economic sectors, from raw materials and manufacturing, to restaurant, retail and banking services. The data contains information for 347 municipalities and 700 industries classified according to the Código the Actividad Económica (CAE) (<http://www.sii.cl/catastro/codigos.htm>).

# PRESENCE-ABSENCE MATRIX DEFINITION:

For the international trade data set, we define the presences of an industry in a country if that country has exports per capita that are at least 25% of the world average for 5 consecutive years. Formally, we do this following:

$$M_{cp}=1 if \frac{\frac{{EXP}_{cp}}{P_{c}}}{\frac{\sum_{c} {EXP}_{cp}}{\sum_{c} P_{c}}}>0.25 and M_{cp}=0 Otherwise$$

Where *M_cp_* is the presence-absence matrix, *EXP_cp_* are the exports of product *p* by country *c*, and *P_c_* is the population of country *c*. For the domestic tax data, we define as a presence a municipality that has one or more firms filing taxes under that industrial classification. We use a single year in this case.

# *NESTEDNESS METRICS: TEMPERATURE AND NODF*

We calculate the nestedness of the exports per capita absence-presence matrices using both, Atmar and Patterson’s temperature metric and Almeida-Neto et al.’s NODF metric. Preparation of these matrices for both analyses is similar. For the temperature metric, the rows and columns of a matrix are sorted and rank-ordered to yield a nested matrix with the absolute minimum temperature possible for this matrix. For the NODF metric, the rows and the columns of a matrix are swapped and rank-ordered by the sum of the presences in each of these rows and columns, respectively. The transformed matrices are then ready to be processed by the following algorithms. For a more detailed explanation, please reference the respective works of Atmar and Patterson (1993) and Almeida-Neto et al. (2008). Also the review by Ulrich, Almeida and Gotelli (2009) is a good place to learn about both of these metrics.

## *ATMAR AND PATTERSON’S TEMPERATURE MEASURE*

Atmar and Patterson's temperature metric calculates the number and the degree of unexpected presences and absences in an ordered adjacency matrix. Unexpected presences and absences are calculated with respect to an *extinction line* that separates the adjacency matrix into two areas: The top-left triangle, which we will call Section 1, where only presences are expected to appear, and the bottom right triangle, which we call Section 2, where only absences are expected (Figure SM2). In a perfectly nested matrix an ideal extinction line is a skew diagonal bisecting the matrix, where all of the presences are to one side of the line and all of the absences are to the other side.

**Figure SM2:** A Perfectly Nested Matrix with M Rows and N columns

Presences in Section 1 that are closer to the extinction line are considered more likely to face extinction. A presence in Section 2, on the other hand, is considered an unexpected presence. Thus, the distance from the extinction line captures the degree of unexpectedness of presence. Conversely, absences in Section 1 are considered unexpected absences.

A perfectly nested matrix is characterized by a temperature of zero degrees. Alternatively, a fully disordered matrix is characterized by a temperature of 100 degrees.

The degree of “unexpectedness” for any presence or absence is the squared ratio of its distance, *d_ij_*, to the ideal extinction line, *D_ij_*. This local unexpectedness is expressed as:

$$u_{ij}=\left( \frac{d_{ij}}{D_{ij}} \right)^{2}$$

The degree of unexpectedness for the matrix, *U*, is the sum of each of these local unexpectedness values. This sum is normalized by the number of rows (*m*) and columns (*n*), to ensure the measure is unaffected by the size or the shape of the adjacency matrix:

$$U= \frac{1}{mn} \sum_{ij} u_{ij}$$

The total unexpectedness is transformed to a temperature scale using a normalization factor. The temperature scale goes from 0 degrees, corresponding to a perfectly ordered matrix, to 100 degrees, indicating a matrix full with unexpected values:

$$T= \frac{100}{U_{max}}U$$

where *U_max_* is 0.04145.


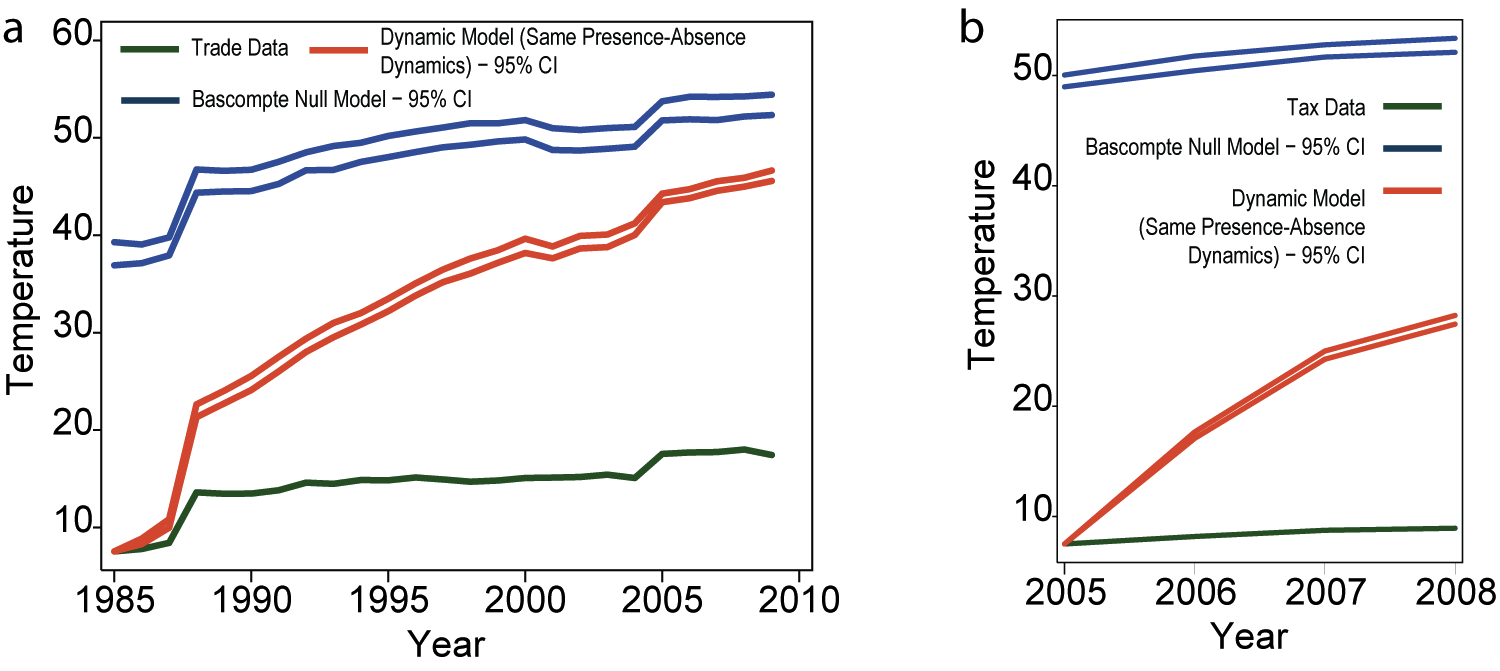
Figure **SM3**: The results shown in Figure 1 f and g of the main text but using temperature.

## *Almeida-Neto et al.’s NODF Measure*

The Nested Overlap and Decreasing Fill, or NODF metric, measures the degree of overlap between an adjacency matrix's rows and columns. NODF is determined by comparing all row-row and all column-column pairs. A row-row pair *ij* is any row *i* paired with each row above it, row *j,* in an ordered matrix. Similarly, a column-column pair *ij* is any column *i* paired with each column behind it, column *j,* in an ordered matrix. This is first achieved by calculating the Paired Overlap, *PO_ij_*, for each row-row and each column-column pair. *PO_ij_* is calculated as the percentage of presences in row or column *i* that are also present in row or column *j*:

$${PO}_{ij}=\frac{\sum O_{ij}}{\sum{MT}_{j}}$$

where *MT_j_* is marginal total, the sum of presences in row or in column *j*, and *O_ij_* is the number of presences overlapping between the row-row or the column-column pair.

 Figure **SM4**: An Ordered Matrix^[[2]](#footnote-2)^

For example, consider rows r1 and r2 from Figure SM4:

Figure **SM4a**: A Sample Row-Row Pair from the Matrix in Figure SM3

In figure SM4a, r2 – the less populated row with three presences – overlaps with two presences in r1 – the more populated row. The *PO_ij_* for the r1-r2 pairing is thus two presences divided by three presences, or *PO_12_* = 66.67%. Similarly for columns, consider figure **SM4b**:

Figure **SM4b**: A Sample Column-Column Pair from the Matrix in Figure **SM4**

Column c4 – the less populated column with only two presences – shares only shared presence with column c1 – the more populated column. Thus, the *PO_ij_* for the c1-c4 pairing is *PO_14_* = 50%.

With the paired overlap, we can now calculate both the decreased fill, *DF_ij,,_* for every row-row and column-column pair. The *DF_ij_* takes one of two values depending on the marginal total, or *MT*, of the rows or the columns in the pair. Thus, in an ordered adjacency matrix, if the marginal total of row *i*, *MT_i_*, is less than the marginal total of row *j*, *MT_j_*, then *DF_ij_* takes on the value of 100. Otherwise, if *MT_i_* is greater than or equal to the *MT_j_*, then *DF_ij_* takes on the value of 0.

$$\left\{ \begin{aligned} {DF}_{ij}=100, &{MT}_{i}<{MT}_{j} \\ {DF}_{ij}=0, &{MT}_{i}\geq{MT}_{j} \end{aligned} \right.$$

The penultimate variable is the paired nestedness, *N_ij_,* for every row-row and every column-column pair. Similar to *DF_ij_*, *N_ij_* can take on only one of two values based on the *DF_ij_* and the *PO_ij_* of its row-row or its column-column pair. Thus, if *DF_ij_* = 100, then *N_ij_* = PO*_ij_*; otherwise, *N_ij_* = 0.

$$\left\{ \begin{aligned} N_{ij}={PO}_{ij}, &{DF}_{ij}=100 \\ N_{ij}=0, & Otherwise \end{aligned} \right.$$

The *N_ij_* is calculated for every row-row and column-column pair in the matrix. The NODF score is the average of all *N_ij_* values:

$$NODF= \frac{\sum N_{ij}}{\frac{n(n-1)}{2}+ \frac{m(m-1)}{2}}$$

where $\frac{n(n-1)}{2}$ and $\frac{m(m-1)}{2}$ are the the total number of possible row-row and column-columns pairs in the matrix. Figure **SM4c** illustrates the entire NODF calculation for the matrix in figure 2.

Figure **SM4c**: The Entire NODF Calculation for the Matrix in Figure 2

# NULL MODELS

## Static Null Model (Bascompte et al.)

Bacompte et al. (2003) introduced a null model to show whether the nested order of the data is statistically meaningful. For this, they introduced a null model (*M_cp_**) in which the probability to find a presence in that same cell of the matrix is equal to the average of the probability of finding it in that row and column in the original matrix (*M_cp_*).

$$P(M_{cp}^{*}=1)= \frac{1}{2}\left( \frac{1}{N_{p}}\sum_{p} M_{cp}+\frac{1}{N_{c}}\sum_{c} M_{cp} \right)$$

Using this model we performed 100 random realizations of the matrix for each year. Then we calculated the Temperature and NODF of each realization of the resulting null matrices to obtain a distribution of possible outcomes. Figures 1f and 1g show the 95% confidence interval Temperature and NODF of these null matrices. Since both the Temperature and NODF of the matrices lie outside the confidence interval, we can say that the nestedness of the matrix is statistically significant.

## Dynamic Null Model

To show that nestedness of the network connecting countries to the products is conserved over time we introduce a dynamic null model. This dynamic null model preserves the exact density of the network and also the number of links that appeared and disappeared each year in each country and each product. First, we calculate the number of links that appeared and disappeared for each year. Then, starting with data for the year 1985, we introduced the same number of appearances and disappearances that were observed in the transition between 1985 and 1986 with a location in the matrix determined by the Bascompte et al. null model explained above. The result is a matrix for year 1986 that has the same density of the real data. We continue this procedure to the last year of our data. The procedure was repeated 100 times, and for each matrix we calculated the Temperature and NODF. Figures 1f and 1g of the main text show the 95% confidence interval of the distribution of Temperature and NODF of these dynamic null matrices. The figures show that the dynamic null model does not keep the same level of order of the real data and disorders rather quickly. Hence, the order of the real-data remains highly nested despite large changes in the links of the network.

# DIVERSITY AND UBIQUITY LINES, AND DISTANCE OF EVENTS

To gauge the position of appearances and disappearances in the presence absence matrix, we introduce the diversity and ubiquity lines as a line indicating where presences would be expected to end if the matrix were to be perfectly nested.

In an adjacency matrix sorted by the sum of its rows and columns, the diversity line is a line that goes through the column that is equal to the number of presences in that row. In the case of locations (countries or municipalities) this is equal to their diversity. For each column, the ubiquity line is one that goes through the row equal to its number of presences. In the case of an industry, this represents its ubiquity, or the number of locations where it is present. Figure **SM5** illustrates the diversity and ubiquity lines, and the distance of an event to them.


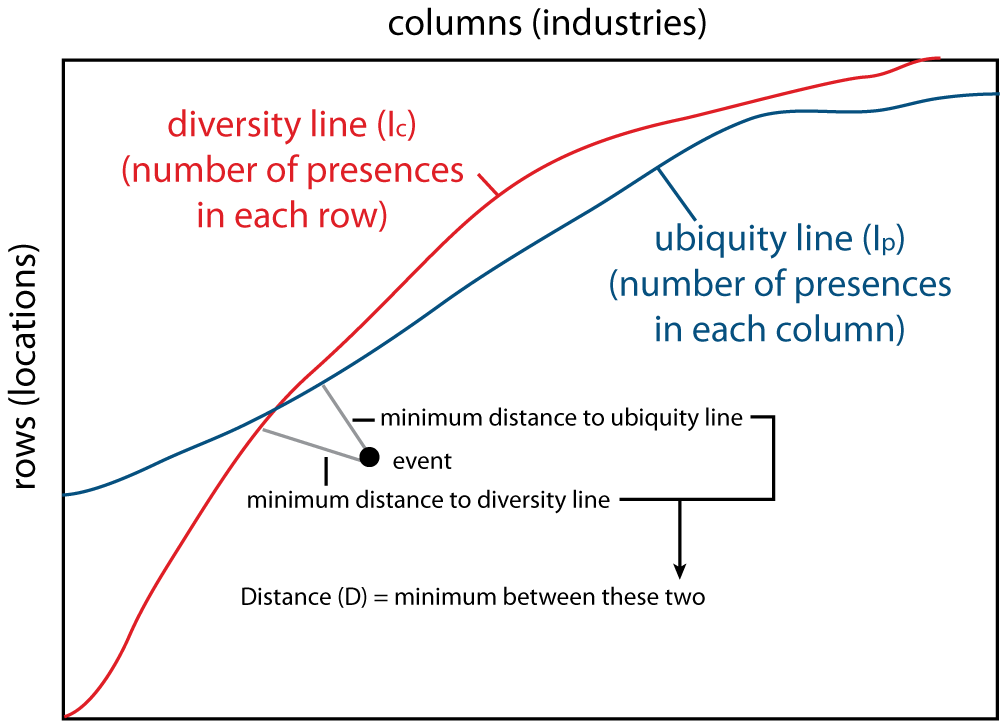

Figure **SM5**: Diversity and Ubiquity lines and the distance of an event to them.

# ROBUSTNESS CHECKS FOR NESTEDNESS

In this section we show the robustness of some of the main stylized facts of the paper to a different definition of presences and absences. Here, we indicate presences and absences using Balassa's (1986) definition of Revealed Comparative Advantage (RCA). Moreover, we use data for all years (1962-2009).

Balassa's (1986) RCA compares the share of a country's exports that a product represents with the share of world trade represented by that same product. If that product represents a share of that country's export that is larger than its share of world trade, then we say that the country has RCA in that product. We define a presence as having RCA≥1 in a product for at least five consecutive years. Figure SM6 a shows the increase in the number of links in the presence-absence matrix of the RCA network between 1966 and 2009. Figure SM6 b shows the RCA country-product network and their respective diversity and ubiquity line for the year 2000. This matrix is characterized by a temperature of 12±2 and a NODF of 21±8. Figure SM6 c shows its respective Bascompte et al. (2003) null model. In this case, temperature is 12±2 and NODF is 21±8.

***
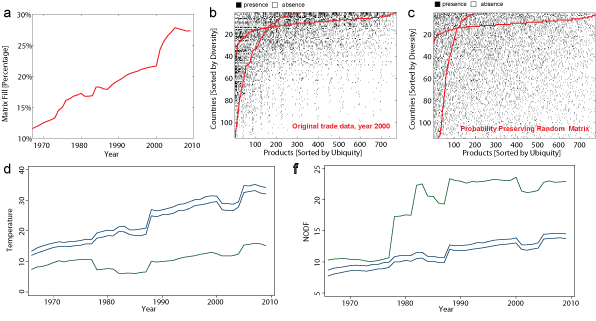
***

**Figure SM6** The nestedness of international economies using RCA. **a** Evolution of the density of the country-product network between 1985 and 2009. **b** Country-product network for the year 2000. **c** Bascompte el al. null model for the matrix shown in **b**. **d** Evolution of the temperature of the country-product network between 1966 and 2009 (green), its corresponding Bascompte et al. null model (blue, upper and lower lines indicate 95% conf. intervals). **f** Same as **d** but using NODF.

Figure SM7 reproduces Figure 2 of the paper's main text using Balassa's (1986) definition of RCA. These figures illustrate the robustness of the analysis to the difference in definition. It is worth noting that using Balassa's (1986) definition of RCA, instead of the exports per capita definition used in the main text, provides slightly weaker, albeit statistically significant, predictions.

***
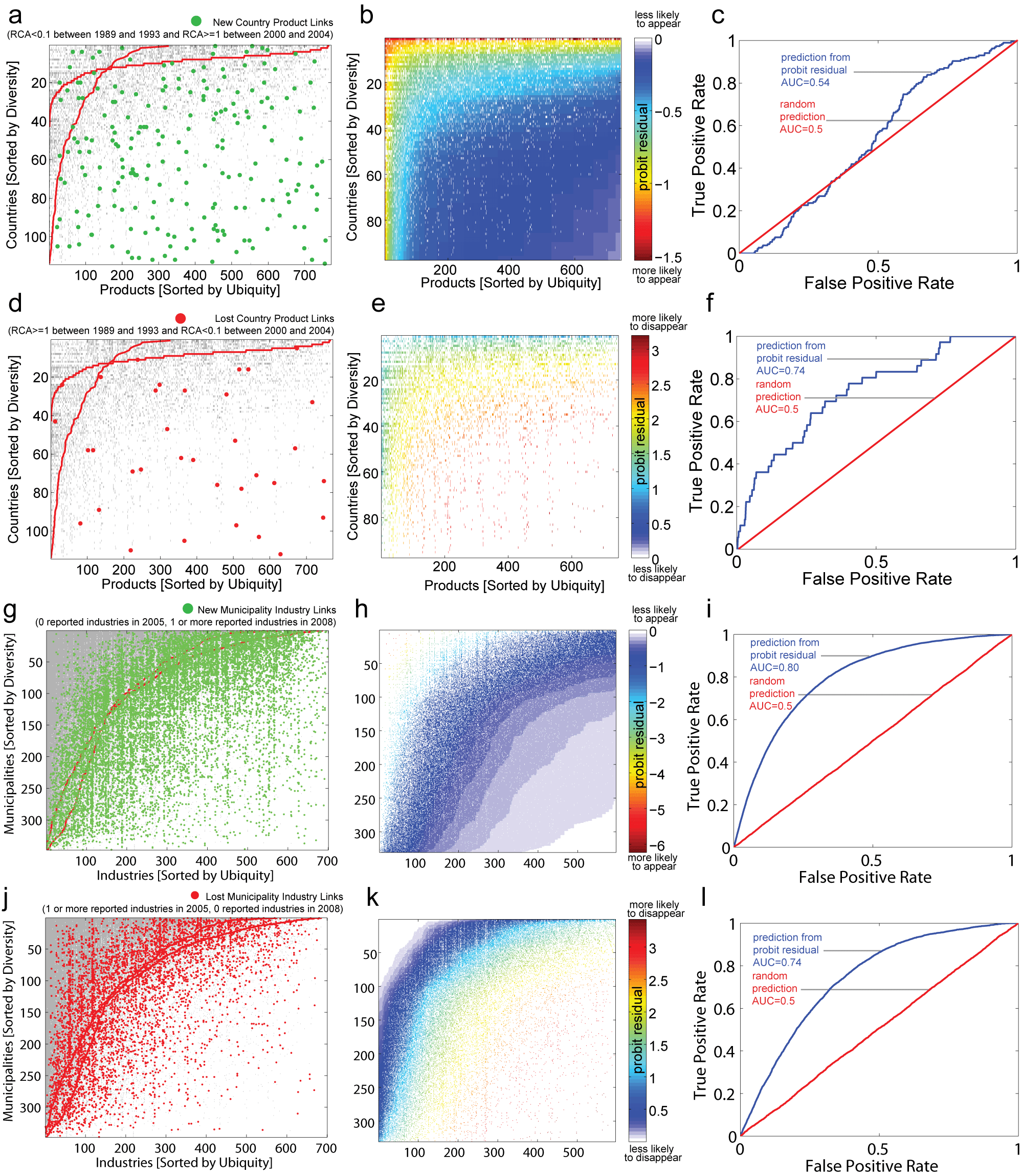
*Figure SM7** Nestedness using RCA. **a** The country-product network for the year 1993 is shown in grey. Green dots show the location of industries that were observed to appear between 1993 and 2000. **d** Same as **a**, but with the industries that disappeared in that period shown in Orange. **b and e** Deviance residuals of the regression presented in (1) of the main text applied to the presences-absences shown in **a-d**. **c and f** ROC curves summarizing the ability of the deviance residuals shown in **b-e**, to predict the appearances and disappearances highlighted in **a and d**.

Finally, Figure SM8 reproduces figure 3 of the main text using Balassa's (1986) RCA to indicate presences and using data for all years. Here, we see that results hold except when the years 1974-1977 are used as predictors. This is because of a large discontinuity in the data classification introduced between 1973 and 1974. This is documented in the second supplementary material of the paper, which shows the fraction of countries that had >0 exports in each product category for all years for the 1006 product categories in the SITC4 rev2 classification.

***
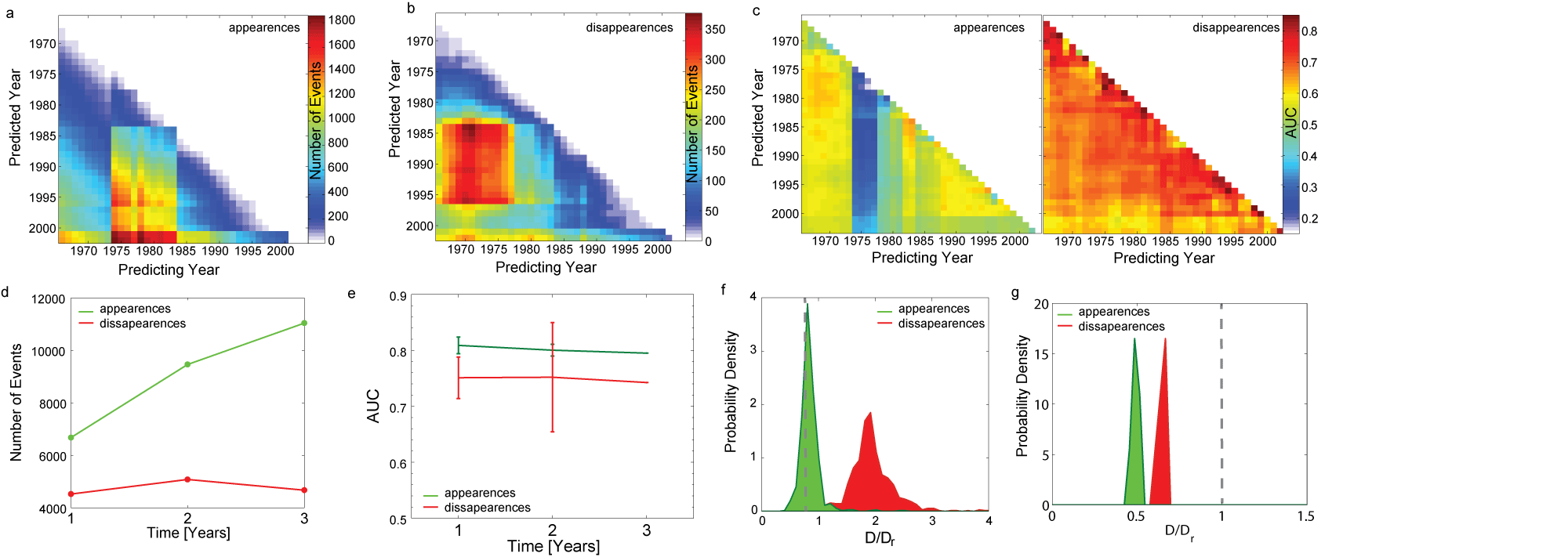
***
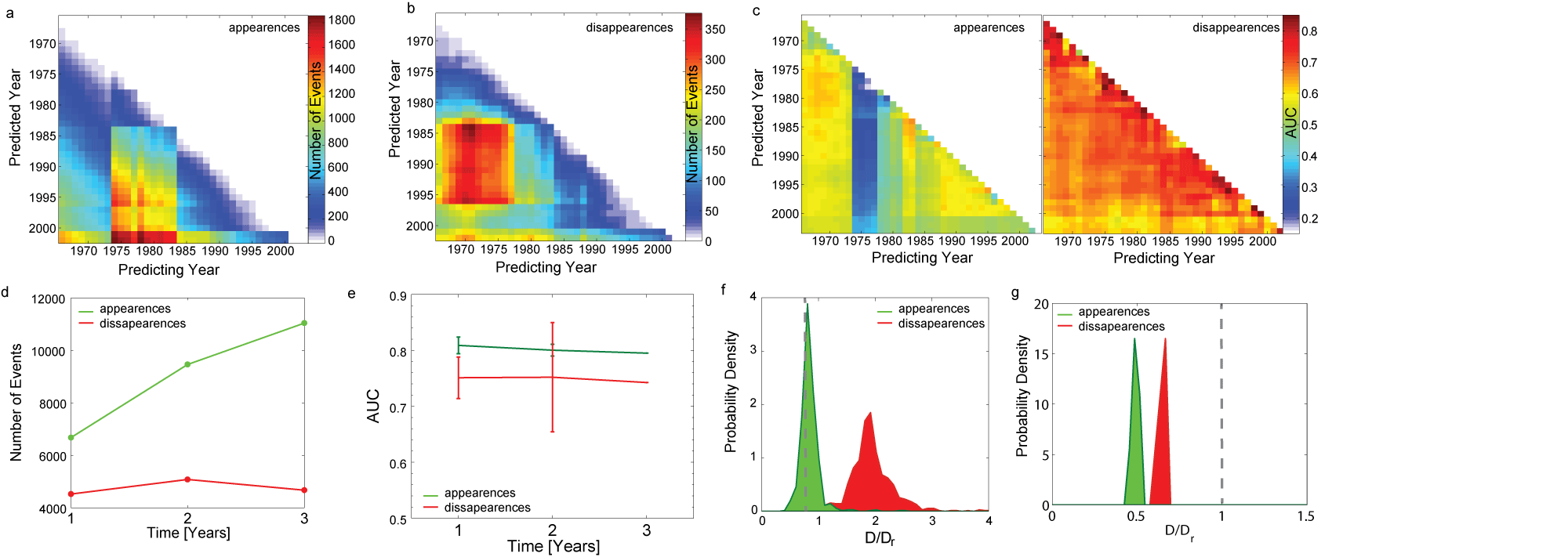


**Figure SM8** Predicting appearances and disappearances using nestedness. **a** Number of appearances for every pair of years in the country-product network. **b** Number of disappearances for every pair of years for the country-product network. **c** Accuracy of the predictions for each pair of years measured using the Area Under the ROC Curve (AUC).

**References:**

Almeida-Neto M, Guimaraes P, Guimaraes PR, Loyola RD, Ulrich W (2008) A consistent metric for nestedness analysis in ecological systems: reconciling concept and measurement. Oikos 117, 1227-1239, doi:10.1111/j.2008.0030-1299.16644.x

Atmar W, Patterson BD (1993) The measure of order and disorder in the distribution of species in fragmented habitat Oecologia 96, 373-382, doi:10.1007/bf00317508

Balassa B (1986) Comparative advantage in manufactured goods – A reappraisal. Review of Economics and Statistics 68, 315-319

Bascompte J, Jordano P, Melian CJ, Olesen, JM (2003) The nested assembly of plant-animal mutualistic networks. Proceedings of the National Academy of Sciences of the United States of America 100, 9383-9387, doi:10.1073/pnas.1633576100

Feenstra RR, Lipsey H, Deng A, and Mo H (2005) “World Trade Flows: 1962-2000” NBER working paper 11040. National Bureau of Economic Research, Cambridge MA.

Ulrich W, Almeida M, Gotelli NJ (2009) A consumer's guide to nestedness analysis. *Oikos* **118**, 3-17, doi:10.1111/j.1600-0706.2008.17053.x

1. For more information visit <http://unstats.un.org/unsd/cr/registry/regcst.asp?Cl=8&Lg=1> [↑](#footnote-ref-1)
2. Figures SM3, SM3a, SM3b, and SM4 are taken directly from: *Almeida-Neto M, Guimaraes P, Guimaraes PR, Loyola RD, Ulrich WA (2008) consistent metric for nestedness analysis in ecological systems: reconciling concept and measurement. Oikos* ***117****, 1227-1239, doi:10.1111/j.2008.0030-1299.16644.x* [↑](#footnote-ref-2)
